# The association between inter-twin birth weight discordance and hepatitis C: The United States 2011–2015 twin birth registration data

**DOI:** 10.1371/journal.pone.0211683

**Published:** 2019-01-30

**Authors:** Yanni Xiao, Minxue Shen, Shujuan Ma, Shi Wu Wen, Hongzhuan Tan

**Affiliations:** 1 Department of Epidemiology and Health Statistics, Xiangya School of Public Health, Central South University, Changsha, Hunan, China; 2 Hunan University of Medicine, Huaihua, Hunan, China; 3 Department of Dermatology, Xiangya Hospital, Central South University, Changsha, Hunan, China; 4 OMNI Research Group, Department of Obstetrics and Gynecology, Faculty of Medicine, University of Ottawa, Ottawa, Canada; 5 Ottawa Hospital Research Institute, Clinical Epidemiology Program, University of Ottawa, Ottawa, Canada; 6 School of Epidemiology, Public Health, and Preventive Medicine, University of Ottawa, Ottawa, Canada; University of Tsukuba, JAPAN

## Abstract

**Background:**

Twins with discordant growth have increased risks of perinatal mortality and morbidity. Previous studies have identified a number of risk factors for inter-twin birth weight discordance, yet no study has examined the effect of maternal hepatitis C infection.

**Methods:**

We used the twin birth records extracted from the 2011 to 2015 United States birth records created by the Centers for Disease Control and Prevention. The outcome variable of this study was inter-twin birth weight discordance, defined as [(birth weight of larger twin–birth weight of smaller twin) / birth weight of larger twin]. The independent association of hepatitis C infection with birth weight discordance was examined using the gamma regression or log binomial regression, adjusted by potential confounders.

**Results:**

Of the 270,256 twin pairs included in the final analysis, 850 (0.31%) had positive hepatitis C. Compared to mothers without hepatitis C, mothers with hepatitis C positive tended to have higher risk of birth weight discordance, but with no statistical significance. After adjustment for potential confounding factors, hepatitis C positive became a significant risk factor for birth weight discordance >25% (relative risk 1.14, 95% confidence interval 1.02−1.29). Sensitivity analyses (by treating birth weight discordance as a continuous outcome or dichotomizing into by different cutoffs) yielded similar results, with relative risks ranging from 1.07 to 1.12 (all *P*<0.05).

**Conclusion:**

Maternal hepatitis C positive is associated with inter-twin birth weight discordance, an important adverse infant outcome in twin pregnancies, although the effect size is small.

## Introduction

Approximately 16% of twin pregnancies have birth weight discordance of at least 20% [1]. Compared with twins with birth weight concordance, twins with discordant growth have increased risks of perinatal mortality and morbidity [1]. Identification of risk factors of inter-twin birth weight discordance is helpful in obstetric care of these pregnancies.

In the past two decades, a number of epidemiologic studies have been conducted to examine risk factors of inter-twin birth weight discordance, and have identified a number of risk factors including advanced maternal age, the use of assisted reproductive technology, hypertensive disorders in pregnancy, and smoking [2–12].

Hepatitis C (HCV) affects about 3% of the world's population, with the highest prevalence in population under 40 years of age [13]. The prevalence of HCV in pregnant women varies with geographical distribution (highest in developing countries), and the prevalence increases in sub-populations of women at high risk for blood-transmitted infections [13]. HCV infection in pregnancy is associated with increased risks of adverse perinatal outcomes such as low birth weight, small for gestational age, need for assisted ventilation, and admission to neonatal intensive care unit [14–18]. Theoretically, HCV infection in pregnancy may increase the risk of birth weight discordance in twins as well. However, no study has examined the effect of HCV infection in pregnancy on inter-twin birth weight discordance. We have therefore analyzed the association between HCV infection in pregnancy and inter-twin birth weight discordance, using a large birth registration data in the United States.

## Methods

### Materials and participants

We used the 2011 to 2015 multiple birth files of the United States created by the Centers for Disease Control and Prevention. The multiple births files contain all twin births in the United States from 2011 to 2015, with data extracted from birth certificates and discharge abstracts. The data is open-access and available on CDC’s official site (https://www.cdc.gov/nchs/data_access/vitalstatsonline.htm). The data has been approved to be used for statistical analysis and report under restrictions for protecting individual person’s information (https://www.cdc.gov/nchs/data_access/restrictions.htm). We restricted our analysis to twin births, with twin set as the unit of analysis. Sets of twins were matched by plurality, year and month of last normal menstrual period, number of prenatal visits, maternal age, mother’s race, mother’s educational level, mother’s body mass index, weight gain during pregnancy, obstetric estimation of gestational age, and year and month of delivery (S1 File). The matching was successful for 90.2% of the twin sets. Twin sets with incomplete match, or any twin being stillbirth, or any twin being less than 300 grams, or any twin with missed information on birth weight, gestational age, or HCV infection were excluded.

The outcome variable of this study was birth weight discordance, which was defined as [(birth weight of larger twin–birth weight of smaller twin) / birth weight of larger twin] × 100%. The confounding factors considered in this study included maternal age, race, educational level, the Special Supplemental Nutrition Program for Women, Infants, and Children (WIC), parity, assisted reproductive technology (ART), diseases prior to or developed during pregnancy (diabetes, gestational diabetes, chronic hypertension, gestational hypertension, eclampsia), body mass index (BMI, kg/m^2^), weight gain during pregnancy, hepatitis B, sexually transmitted diseases (STDs), gender of infants, and congenital anomalies.

### Statistical method

Demographic and clinical characteristics of the study population were described and compared between women with HCV and those without. Means and standard deviations were used to describe continuous variables, and differences were tested using ANOVA. Counts and frequencies were used to describe categorical variables, and differences were tested using chi-square tests. Twin birth weight discordance were described and compared between women with HCV and those without. We used either continuous or dichotomized discordance by cutoff values of 15%, 20%, or 25%.

All potential confounding factors associated with HCV infection and birth weight discordance were included in the adjusted models. We used generalized linear models to examine the independent association of HCV infection with birth weight discordance. For dichotomized discordance, log binomial regression was used; while for continuous discordance, Gamma regression was used. General linear model was not used in our study because of the poor model fit attributed to the skewed distribution of birth weight discordance. Adjusted regression coefficient (*β*) or relative risk (RR) were used to represent the effect size of HCV infection.

In the adjusted models, confounding factors were categorized as the followings: maternal age<25, 25−34, and ≥35, BMI underweight (<18.5 kg/m^2^), normal (18.5−24.9 kg/m^2^), overweight (25.0−29.9 kg/m^2^), and obese (≥30.0 kg/m^2^); weight gain during pregnant was divided by tertile: 1^st^ tertile (<30 pounds), 2^nd^ tertile (30−42 pounds), and 3^rd^ tertile (≥43 pounds). Some of the mother’s education and race groups in the original files were combined in order to enhance the power and robustness of estimation. All reported congenital anomalies (including anencephaly, spina bifida, congenital heart disease, congenital diaphragmatic hernia, omphalocele, gastroschisis, limb reduction defect, cleft lip, cleft palate, hypospadias, Down Syndrome, and suspected chromosomal disorder) were combined as a single variable. Twin match and data analyses were performed using the software of SAS version 9.4 (SAS Institute Inc., Cary, North Carolina, USA).

## Results

**Fig 1:**
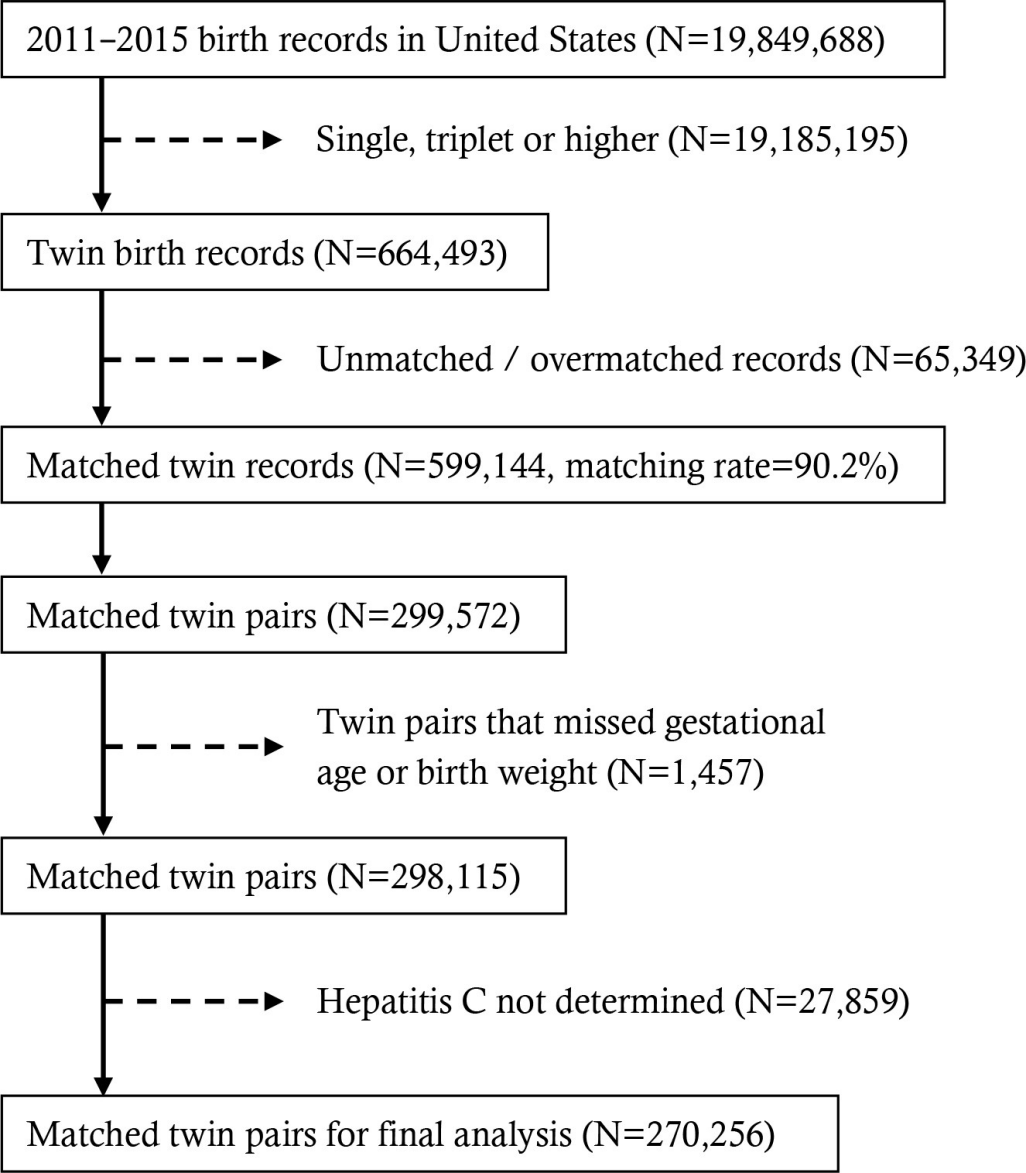
Flow chart of object selection.

There were 664,490 twin records from 2011 to 2015 in the original files. Of these, 599,141 (90.2%) were successfully matched. After excluding records with missing information on outcome and critical co-variables, 540,512 twin records (or 270,256 pairs of twins) were included in the final analysis (Fig 1). The mean age of the included (HCV reported) and excluded women (HCV not reported, 9.3%) were 30.2 ± 6.0 and 30.4 ± 6.1 years, respectively; and the mean BMI of the two groups were 27.1 ± 6.9 and 27.5± 7.4 kg/m^2^, respectively. No major selection bias existed; as a result, imputation for missing data was not applied.

Of the 270,256 twin pairs, 850 mothers (0.31%) had positive HCV infection. Compared to mothers without HCV, mothers with HCV infection tended to be younger, more likely being white, with lower education, more likely being WIC recipients, less likely to use ART to conceive, more likely being multiparous, with lower BMI and gained less weight during pregnancy, with STDs and hepatitis B infections, shorter gestational age, lower total birth weight (combined birth weight of the twins), and higher congenital anomaly rate (Table 1). On the other hand, no difference between the two groups in pre- pregnancy diabetes, gestational diabetes, chronic hypertension, gestational hypertension, eclampsia, and sex of twins was observed.

**Table 1:** Comparison of demographic and clinical characteristics between those pregnant women with hepatitis C and those without, United States, 2011−2015.

	With hepatitis C (n = 850)	Without hepatitis C (n = 269,406)	*P*
Maternal age (years)	29.0± 5.0	30.2± 6.0	<0.001
<25	179 (21.1)	50,050 (18.6)	<0.001
25−34	546 (64.2)	155,255 (57.6)	
≥35	125 (14.7)	64,101 (23.8)	
Mother’s education			
Associate degree and below	771 (90.7)	157,002 (58.3)	<0.001
Bachelor degree and above	64 (7.5)	108,199 (40.2)	
Unknown	15 (1.8)	4,205 (1.6)	
Mother’s Race			
White	722 (84.9)	200,531 (74.4)	<0.001
Black	80 (9.4)	46,122 (17.1)	
AIAN	17 (2.0)	1,855 (0.7)	
Asian and pacific islander	9 (1.1)	16,181 (6.0)	
Multiple race	22 (2.6)	4,717 (1.8)	
Mother’s BMI (kg/m^2^)	26.3± 6.4	27.1± 6.9	0.002
Underweight (<18.5)	34 (4.0)	7,318 (2.7)	0.015
Normal (18.5−24.9)	388 (45.6)	114,443 (42.5)	
Overweight (25.0−29.9)	185 (21.8)	65,513 (24.3)	
Obese (≥30)	202 (23.8)	70.924 (26.3)	
Unknown	41 (4.8)	11,208 (4.2)	
Weight gain (pounds)	35.6± 18.8	37.0± 17.3	0.017
1^st^tertile (<30)	307 (36.1)	82,420 (30.6)	<0.001
2^nd^tertile (30−42)	224 (26.4)	82,091 (30.5)	
3^rd^tertile (≥43)	255 (30.0)	90,761 (33.7)	
Unknown	64 (7.5)	14,134 (5.2)	
Parity			
Primipara	160 (18.8)	101,491 (37.7)	<0.001
Multipara	690 (81.2)	167,915 (62.3)	
WIC	547 (64.4)	98,761 (36.7)	<0.001
Use of ART	16 (1.9)	24,218 (9.2)	<0.001
Previous diabetes	11 (1.3)	2,416 (0.9)	0.218
Gestational diabetes	56 (6.6)	20,446 (7.6)	0.276
Chronic hypertension	22 (2.6)	6,162 (2.3)	0.553
Gestational hypertension	77 (9.1)	29,537 (11.0)	0.078
Eclampsia	3 (0.4)	1,654 (0.6)	0.332
STDs ^a^	36 (4.2)	4,075 (1.5)	<0.001
Hepatitis B	66 (7.8)	493 (0.2)	<0.001
Twin sex			
Same	540 (63.5)	172,298 (64.0)	0.796
Different	310 (36.5)	97,108 (36.0)	
Gestational age (weeks)	35.1± 3.4	35.4 ± 3.4	0.001
Total birth weight (grams)	4,338± 1,135	4,719± 1,160	<0.001
Congenital anomaly ^b^	11 (1.3)	1937 (0.7)	0.048

BMI: body mass index. WIC: the Special Supplemental Nutrition Program for Women, Infants, and Children. ART: assisted reproductive technology. STDs: sexually transmitted diseases.

^a^ STDs included gonorrhea, syphilis, and chlamydia.

^b^ Congenital anomalies included anencephaly, spina bifida, congenital heart disease, congenital diaphragmatic hernia, omphalocele, gastroschisis, limb reduction defect, cleft lip, cleft palate, hypospadias, Down Syndrome, and suspected chromosomal disorder.

Table 2 compares birth weight discordance between the two groups. Twins born to mothers with HCV had significantly higher discordance as compared with twins born to mothers without.

**Table 2:** Comparison of distribution of inter-twin birth weight discordance (%) between those pregnant women with hepatitis C and those without, United States, 2011−2015.

Inter-twin birth weight discordance	With hepatitis C (n = 850)	Without hepatitis C (n = 269,406)	*P*
Range	0.0−60.9	0.0−88.9	
Median (IQR)	9.5 (12.5)	8.8 (11.5)	
Mean (SD)	11.6± 9.6	10.9± 9.1	0.039
Discordance > 15%	245 (28.8)	71,453 (26.5)	0.129
Discordance > 20%	142 (16.7)	39,396 (14.6)	0.086
Discordance > 25%	81 (9.5)	20,974 (7.8)	0.058

SD: standard deviation. IQR: interquartile range.

After adjustment for potential confounding factors, as shown in Table 3, maternal HCV infection was a significant risk factor for birth weight discordance, with RRs ranging from 1.07 to 1.14 under different cutoffs (all *P* values <0.05).

**Table 3:** The effect size of hepatitis C infection on inter-twin birth weight discordance.

Model	Crude estimates (95%CI)	Adjusted estimates (95%CI) ^b^
Continuous discordance ^a^	1.06 (0.99, 2.89)	1.07 (1.01, 1.13)
Discordance >15%	1.08 (0.98, 2.97)	1.10 (1.01, 1.21)
Discordance >20%	1.09 (0.98, 3.01)	1.12 (1.01, 1.23)
Discordance >25%	1.12 (0.99, 3.05)	1.14 (1.02, 1.29)

^a^ Continuous discordance was modeled using Gama regression. Dichotomized discordance was modeled using log binomial regression. Confounders in all models were identical.

^b^ Adjusted confounders including maternal age, education, race, BMI, weight gain, parity, WIC, use of ART, STDs, hepatitis B, and congenital anomalies. For the continuous outcome, the estimate (regression coefficient) meant that the mean birth weight discordance among women with hepatitis C was X times to that among those without hepatitis C. For the dichotomized outcome, the estimate (relative risk) meant that the probability of having twin birth weight discordance among women with hepatitis C was X times to that among those without hepatitis C.

## Discussion

Our study based on a large cohort of twins in the United States demonstrated that twins born to mothers with HCV infection had significantly higher risk of developing inter-twin birth weight discordance than twins born to mothers without HCV infection, after adjusting for a number of known risk factors of birth weight discordance. This association remained essentially the same using different cut-off points or treating birth weight discordance as a continuous outcome variable. To our knowledge, this is the first study that has examined the association between maternal HCV infection and inter-twin birth weight discordance. The large sample of our study allowed a stable estimation and adjustment for a number of risk factors simultaneously. The association between maternal HCV infection and inter-twin birth weight discordance became stronger and statistically significant after adjusting for a number of confounding factors, including maternal demographic factors. We acknowledged that the effect size of HCV infection on birthweight discordance was not very large for clinical importance. However, it is still a problem, from scientific point of view, that may still worth exploration.

A few studies have assessed the association between maternal HCV and infant outcomes other than inter-twin birth weight discordance [14–18]. Earlier studies with small samples yielded inconsistent results [15–18]. A recent large population-based cohort study of singleton births found that compared with infants born to HCV negative mothers, infants born to HCV positive mothers were at increased risks of low birth weight, small for gestational age, need assisted ventilation, and to require neonatal intensive care unit admission [14]. These findings were consistent with our results in twin births. The HCV positive rate in the large cohort of singleton births in Washington State found a HCV positive rate of 0.20% [14], which was slightly lower than 0.31% in the twin births observed in our study. Altogether, these study results suggest that maternal HCV may have negative impact on infant health, both in singletons and twins. However, the mechanism of maternal HCV infection on infant outcomes is not understood. Of note is that the pathophysiology of discordant growth is different in monochorionic and dichorionic twin pregnancies. While the discordant growth in dichorionic twin pregnancy is mainly caused by discordant placental size and function, in monochorionic twin pregnancy the magnitude of discordant growth is influenced not only by abnormal placental sharing but also by the direction of blood flow interchange through the placental anastomoses [19, 20]. Because the data could not distinguish the type of twins, we were not able to propose an explicit hypothesis for the mechanism for discordant growth.

Our study has limitations. Because universal HCV testing in pregnancy is not mandatory in the United States, mothers recorded with HCV positive may have increased risks for care providers to initiate test, which could introduce ascertainment bias. However, there is no data reporting the rate of antenatal HCV test. According to a previous report, the rate of HCV screening was 4.31% in population aged above 8 years old [21]. During 2009–2014, the prevalence of maternal HCV infection among reporting states in the U.S. increased from 1.8 to 3.4 per 1,000 live births [22]. According to 2017 CDC data, the HCV antibody positive rate in tests sent from obstetricians was 0.37% [23], far lower than any other provider groups (pediatrics, 1%; primary care, 1.62%; infectious diseases, 6.57%; and gastroenterology, 14.69%). Currently, risk-based screening is recommended; and the fact that the HCV prevalence is significantly lower in pregnant women than other population is possibly because HCV infected patients are not being questioned, and therefore not being identified. Mothers from low social economic status whom tended to have more health problems, had higher HCV positive rate. On the other hand, white mothers, whom tended to have fewer health problems than those of other racial groups, also had higher HCV positive rate. This further complicates the situation, and suggests that potential ascertainment bias from sources other than increased risks for care providers to initiate test may also play a role in HCV positive. However, the association became stronger and statistically significant after adjusting a number of confounding factors including mother’s race and social demographic factors, suggesting that the observed bias/confounding could not explain away of the association. The 0.31% rate of HCV positive rate noted in the birth certificates was low, suggesting that some HCV positive mothers may have been misclassified as the HCV negative group. About 10% women missed information on HCV status. Because the missing was not random and for women with missing HCV status, most of the co-variables such as hepatitis B infection, STD, smoking etc. were missing as well, we could not do imputation for missing HCV status. Fortunately, for the few available co-variables that the women with missing HCV status, such as age and BMI, the distribution between the included (N = 270,256) and excluded (N = 27,859) was similar, suggesting no major bias/confounding was introduced by missing HCV status. Although we have employed rigorous measures to control confounding, some potential confounding factors may not be measured and collected into the database. As a result, residual confounding may still exist. Another limitation is that the birth certificates documented HCV infection cannot differentiate those with viremia and those without. As a result, we could not examine the direct effect of replicating virus on the outcome.

HCV infection in pregnancy represents a non-negligible health problem for unborn fetus, and our study adds to the literature by highlighting its potential impact on inter-twin birth weight discordance, an important adverse outcome to be prevented in twin pregnancies. Regardless the mechanisms, the increased risk of inter-twin birth weight discordance for twins born to these mothers suggests that extra attention should be paid in the care of them. To prevent transmission of HCV, it is important that health care providers adhere to standard precautions, follow fundamental infection-control principles, and use appropriate procedural techniques. Because no safe and effective method exists to reduce vertical transmission of HCV once a woman becomes pregnant, it is important to identify and treat HCV before pregnancy [13, 24]. In summary, we advocate a universal screening rather than risk-based screening for HCV in women of childbearing age.

## Supporting information

S1 File SAS code for twin matching and data cleaning. (PDF)
