# Supplementary material for: The association between inter-twin birth weight discordance and hepatitis C: The United States 2011–2015 twin birth registration data
Source: PLoS One. 2019 Jan 30;14(1):e0211683. doi: 10.1371/journal.pone.0211683 (PMC6353199; doi:10.1371/journal.pone.0211683)
Supplement: S1 File — (PDF) [file pone.0211683.s001.pdf]

```
/*************************************
|    US Twin Birth 2011-2015       |
|    Data clean and twin matching   |
|    Programer: Dr. Minxue Shen     |
*************************************/

libname XIAO 'D:\SAS_Temp';

/**All original data were downloaded from the U.S. CDC offical website/
/***Variables in data from 2011 to 2015 are not identical***/
/*2011 Data*/
data work.US2011;
infile 'D:\SAS_Temp\Nat2011.txt';
input DOB_YY 15-18 DOB_MM 19-20 DOB_WK 29 MAGER 89-90 RESTATUS 138
     MRACE15 139-140 HISPANIC_M 148 DMAR 153 MEDUC 155 FAGECOMB 182-183
FRACE15 188-189
     FRACE6 191 HISPANIC_F 195 FEDUC 197 PRIORTERM 208-209 LBO_REC 212
TBO_REC 217
     ILLB_R 534-536 ILOP_R 539-541 ILP_R 544-546 PRECARE 245-246 PREVIS
270-271
     WIC $ 281 CIG_0 282-283 CIG_1 284-285 CIG_2 286-287 CIG_3 288-289
     M_Ht_In 555-556 BMI 529-532 PWgt_R 549-551 DWgt_R 299-301 WTGAIN
276-277
     RF_PDIAB $ 313 RF_GDIAB $ 314 RF_PHYPE $ 315 RF_GHYPE $ 316
RF_EHYPE $ 317 RF_PPTERM $ 318
     RF_INFTR $ 321 RF_FEDRG $ 322 RF_ARTEC $ 323 RF_CESAR $ 324
RF_CESARN 325-326
     IP_GON $ 345 IP_SYPH $ 346 IP_CHLAM $ 348 IP_HEPB $ 349 IP_HEPC
$ 350
     ME_ROUT 393 DPLURAL 423 IMP_PLUR 425 SETORDER_R 557 SEX $ 436
IMP_SEX 437
     DLMP_YY 442-445 DLMP_MM 438-439 COMBGEST 451-452 DBWT 463-466
     CA_ANEN $ 492 CA_MNSB $ 493 CA_CCHD $ 494 CA_CDH $ 495 CA_OMPH
$ 496 CA_GAST $ 497
     CA_LIMB $ 498 CA_CLEFT $ 499 CA_CLPAL $ 500 CA_DOWNS $ 501
CA_DISOR $ 502 CA_HYPO $ 503;
run;

/*2012 Data*/
data work.US2012;
infile 'D:\SAS_Temp\Nat2012.txt';
input DOB_YY 15-18 DOB_MM 19-20 DOB_WK 29 MAGER 89-90 RESTATUS 138
     MRACE15 139-140 HISPANIC_M 148 DMAR 153 MEDUC 155 FAGECOMB 182-183
FRACE15 188-189
     FRACE6 191 HISPANIC_F 195 FEDUC 197 PRIORTERM 208-209 LBO_REC 212
TBO_REC 217
     ILLB_R 534-536 ILOP_R 539-541 ILP_R 544-546 PRECARE 245-246 PREVIS
270-271
     WIC $ 281 CIG_0 282-283 CIG_1 284-285 CIG_2 286-287 CIG_3 288-289
     M_Ht_In 555-556 BMI 529-532 PWgt_R 549-551 DWgt_R 299-301 WTGAIN
276-277
     RF_PDIAB $ 313 RF_GDIAB $ 314 RF_PHYPE $ 315 RF_GHYPE $ 316
```

```
RF_EHYPE $ 317 RF_PPTERM $ 318
     RF_INFTR $ 321 RF_FEDRG $ 322 RF_ARTEC $ 323 RF_CESAR $ 324
RF_CESARN 325-326
     IP_GON $ 345 IP_SYPH $ 346 IP_CHLAM $ 348 IP_HEPB $ 349 IP_HEPC
$ 350
     ME_ROUT 393 DPLURAL 423 IMP_PLUR 425 SETORDER_R 557 SEX $ 436
IMP_SEX 437
     DLMP_YY 442-445 DLMP_MM 438-439 COMBGEST 451-452 DBWT 463-466
     CA_ANEN $ 492 CA_MNSB $ 493 CA_CCHD $ 494 CA_CDH $ 495 CA_OMPH
$ 496 CA_GAST $ 497
     CA_LIMB $ 498 CA_CLEFT $ 499 CA_CLPAL $ 500 CA_DOWNS $ 501
CA_DISOR $ 502 CA_HYPO $ 503;
run;

/*2013 Data*/
data work.US2013;
infile 'D:\SAS_Temp\Nat2013.txt';
input DOB_YY 15-18 DOB_MM 19-20 DOB_WK 29 MAGER 89-90 RESTATUS 138
     MRACE15 139-140 HISPANIC_M 148 DMAR 153 MEDUC 155 FAGECOMB 182-183
FRACE15 188-189
     FRACE6 191 HISPANIC_F 195 FEDUC 197 PRIORTERM 208-209 LBO_REC 212
TBO_REC 217
     ILLB_R 534-536 ILOP_R 539-541 ILP_R 544-546 PRECARE 245-246 PREVIS
270-271
     WIC $ 281 CIG_0 282-283 CIG_1 284-285 CIG_2 286-287 CIG_3 288-289
     M_Ht_In 555-556 BMI 529-532 PWgt_R 549-551 DWgt_R 299-301 WTGAIN
276-277
     RF_PDIAB $ 313 RF_GDIAB $ 314 RF_PHYPE $ 315 RF_GHYPE $ 316
RF_EHYPE $ 317 RF_PPTERM $ 318
     RF_INFTR $ 321 RF_FEDRG $ 322 RF_ARTEC $ 323 RF_CESAR $ 324
RF_CESARN 325-326
     IP_GON $ 345 IP_SYPH $ 346 IP_CHLAM $ 348 IP_HEPB $ 349 IP_HEPC
$ 350
     ME_ROUT 393 DPLURAL 423 IMP_PLUR 425 SETORDER_R 557 SEX $ 436
IMP_SEX 437
     DLMP_YY 442-445 DLMP_MM 438-439 COMBGEST 451-452 DBWT 463-466
     CA_ANEN $ 492 CA_MNSB $ 493 CA_CCHD $ 494 CA_CDH $ 495 CA_OMPH
$ 496 CA_GAST $ 497
     CA_LIMB $ 498 CA_CLEFT $ 499 CA_CLPAL $ 500 CA_DOWNS $ 501
CA_DISOR $ 502 CA_HYPO $ 503;
run;

/*2014 Data*/
data work.US2014;
infile 'D:\SAS_Temp\Nat2014.txt';
input DOB_YY 9-12 DOB_MM 13-14 DOB_WK 23 MAGER 75-76 RESTATUS 104
     MRACE15 108-109 HISPANIC_M 115 DMAR 120 MEDUC 124 FAGECOMB 147-148
FRACE15 154-155
     FRACE6 153 HISPANIC_F 160 FEDUC 163 PRIORTERM 175-176 LBO_REC 179
TBO_REC 182
     ILLB_R 198-200 ILOP_R 206-208 ILP_R 214-216 PRECARE 224-225 PREVIS
238-239
```

```
     WIC $ 251 CIG_0 253-254 CIG_1 255-256 CIG_2 257-258 CIG_3 259-260
     M_Ht_In 280-281 BMI 283-286 PWgt_R 292-294 DWgt_R 299-301 WTGAIN
304-305
     RF_PDIAB $ 313 RF_GDIAB $ 314 RF_PHYPE $ 315 RF_GHYPE $ 316
RF_EHYPE $ 317
     RF_PPTERM $ 318 RF_INFTR $ 325 RF_FEDRG $ 326 RF_ARTEC $ 327
RF_CESAR $ 331
     RF_CESARN 332-333 IP_GON $ 343 IP_SYPH $ 344 IP_CHLAM $ 345
IP_HEPB $ 346
     IP_HEPC $ 347 ME_ROUT 402 DPLURAL 454 IMP_PLUR 456 SETORDER_R 459
SEX $ 475 IMP_SEX 476
     DLMP_YY 481-484 DLMP_MM 477-478 COMBGEST 490-491 DBWT 504-507
     CA_ANEN $ 537 CA_MNSB $ 538 CA_CCHD $ 539 CA_CDH $ 540 CA_OMPH
$ 541 CA_GAST $ 542
     CA_LIMB $ 549 CA_CLEFT $ 550 CA_CLPAL $ 551 CA_DOWNS $ 552
CA_DISOR $ 553 CA_HYPO $ 554;
run;

/*2015 Data*/
data work.US2015;
infile 'D:\SAS_Temp\Nat2015.txt';
input DOB_YY 9-12 DOB_MM 13-14 DOB_WK 23 MAGER 75-76 RESTATUS 104
     MRACE15 108-109 HISPANIC_M 115 DMAR 120 MEDUC 124 FAGECOMB 147-148
FRACE15 154-155
     FRACE6 153 HISPANIC_F 160 FEDUC 163 PRIORTERM 175-176 LBO_REC 179
TBO_REC 182
     ILLB_R 198-200 ILOP_R 206-208 ILP_R 214-216 PRECARE 224-225 PREVIS
238-239
     WIC $ 251 CIG_0 253-254 CIG_1 255-256 CIG_2 257-258 CIG_3 259-260
     M_Ht_In 280-281 BMI 283-286 PWgt_R 292-294 DWgt_R 299-301 WTGAIN
304-305
     RF_PDIAB $ 313 RF_GDIAB $ 314 RF_PHYPE $ 315 RF_GHYPE $ 316
RF_EHYPE $ 317
     RF_PPTERM $ 318 RF_INFTR $ 325 RF_FEDRG $ 326 RF_ARTEC $ 327
RF_CESAR $ 331
     RF_CESARN 332-333 IP_GON $ 343 IP_SYPH $ 344 IP_CHLAM $ 345
IP_HEPB $ 346
     IP_HEPC $ 347 ME_ROUT 402 DPLURAL 454 IMP_PLUR 456 SETORDER_R 459
SEX $ 475 IMP_SEX 476
     DLMP_YY 481-484 DLMP_MM 477-478 COMBGEST 490-491 DBWT 504-507
     CA_ANEN $ 537 CA_MNSB $ 538 CA_CCHD $ 539 CA_CDH $ 540 CA_OMPH
$ 541 CA_GAST $ 542
     CA_LIMB $ 549 CA_CLEFT $ 550 CA_CLPAL $ 551 CA_DOWNS $ 552
CA_DISOR $ 553 CA_HYPO $ 554;
run;

/*Select twins*/
proc sql;
create table work.US2011twin as
select * from work.US2011 where DPLURAL=2;

create table work.US2012twin as
```

```
select * from work.US2012 where DPLURAL=2;

create table work.US2013twin as
select * from work.US2013 where DPLURAL=2;

create table work.US2014twin as
select * from work.US2014 where DPLURAL=2;

create table work.US2015twin as
select * from work.US2015 where DPLURAL=2;

create table work.combine as
select * from work.US2011twin union
select * from work.US2012twin union
select * from work.US2013twin union
select * from work.US2014twin union
select * from work.US2015twin;
quit;

data work.combine;
set work.combine;
/*Congenital anormalies*/
if CA_ANEN='Y' then CA_ANEN1=1;else CA_ANEN1=0;
if CA_MNSB='Y' then CA_MNSB1=1;else CA_MNSB1=0;
if CA_CCHD='Y' then CA_CCHD1=1;else CA_CCHD1=0;
if CA_CDH='Y'  then CA_CDH1=1; else CA_CDH1=0;
if CA_OMPH='Y' then CA_OMPH1=1;else CA_OMPH1=0;
if CA_GAST='Y' then CA_GAST1=1;else CA_GAST1=0;
if CA_LIMB='Y' then CA_LIMB1=1;else CA_LIMB1=0;
if CA_HYPO='Y' then CA_HYPO1=1;else CA_HYPO1=0;
if CA_CLEFT='Y' then CA_CLEFT1=1;else CA_CLEFT1=0;
if CA_CLPAL='Y' then CA_CLPAL1=1;else CA_CLPAL1=0;
if CA_DOWNS in ('C','P') then CA_DOWNS1=1;else CA_DOWNS1=0;
if CA_DISOR in ('C','P') then CA_DISOR1=1;else CA_DISOR1=0;
run;

/*Identify twins*/
proc sql;
  create table work.mother as
    select distinct DOB_YY,DOB_MM,DOB_WK,MAGER,MEDUC,MRACE15,
           BMI,WTGAIN,DLMP_YY,DLMP_MM,PREVIS,COMBGEST
      from work.combine;
quit;

data work.mother;
set work.mother;
ID=_n_;
run;

/***Matching twins***/
proc sql;
  create table work.LINK as
```

```
    select * from work.mother left join work.combine
      on mother.MAGER=combine.MAGER
      and mother.MEDUC=combine.MEDUC
      and mother.MRACE15=combine.MRACE15
      and mother.BMI=combine.BMI
      and mother.WTGAIN=combine.WTGAIN
      and mother.DLMP_YY=combine.DLMP_YY
      and mother.DLMP_MM=combine.DLMP_MM
      and mother.PREVIS=combine.PREVIS
      and mother.DOB_YY=combine.DOB_YY
      and mother.DOB_MM=combine.DOB_MM
      and mother.COMBGEST=combine.COMBGEST;
quit;

proc sort data=work.LINK;
by ID;
run;

proc sql;
  create table work.a as
    select distinct ID, count(ID) as num
      from work.LINK
      group by ID;
quit;

data work.a;
set work.a;
  if num=1 or num>2 then delete;
run;

proc sql;
  create table work.b as
    select * from work.a left join work.LINK
      on a.ID=LINK.ID;
quit;

data work.b;
set work.b;
if SEX='M' then GENDER=1;
else if SEX='F' then GENDER=0;
run;

/*Data cleaning*/
proc sql;
 create table work.final as
    select ID,DOB_YY as Year, MAGER as MAGE, MEDUC, MRACE15,
HISPANIC_M, BMI,WTGAIN,
         RESTATUS, DMAR as MARRIAGE, FAGECOMB as FAGE, FRACE15, FRACE6,
HISPANIC_F,
         FEDUC, WIC, CIG_0, CIG_1, CIG_2, CIG_3, PREVIS, PRECARE,
         /*adverse outcomes*/
         RF_PDIAB, RF_GDIAB, RF_PHYPE, RF_GHYPE, RF_EHYPE,
```

```
          RF_PPTERM, RF_INFTR, RF_FEDRG, RF_ARTEC, RF_CESAR,
          /*infection*/
          IP_GON, IP_SYPH, IP_CHLAM,IP_HEPB, IP_HEPC,
          /*delivery and infant info*/
          max(ME_ROUT) as DELIVERY, max(COMBGEST) as GA, GENDER,
sum(GENDER) as SEX,
          DBWT, max(DBWT) as maxwt, min(DBWT) as minwt, sum(DBWT) as
sumwt, max(TBO_REC) as TBO,
          /*congenital anomalies*/
          max(CA_ANEN1) as CA_ANEN, max(CA_MNSB1) as CA_MNSB,
max(CA_CCHD1) as CA_CCHD,
          max(CA_CDH1) as CA_CDH, max(CA_OMPH1) as CA_OMPH,
max(CA_GAST1) as CA_GAST,
          max(CA_LIMB1) as CA_LIMB, max(CA_HYPO1) as CA_HYPO,
max(CA_CLEFT1) as CA_CLEFT,
          max(CA_CLPAL1) as CA_CLPAL, max(CA_DOWNS1) as CA_DOWNS,
max(CA_DISOR1) as CA_DISOR
    from work.b group by ID;
quit;

data work.final_1;
set work.final;
if minwt=maxwt then wt_order=1;
else if DBWT=minwt then wt_order=1;
else if DBWT=maxwt then wt_order=2;

if minwt<300 then delete;
if maxwt=9999 then delete;
if minwt=9999 then delete;
if GA=. then delete;
if GA=99 then delete;
if maxwt=. then delete;
if minwt=. then delete;
run;


proc sort data=work.final out=work.USfinal nodupkey;
  by ID;
run;

/*Data recode*/
data work.USfinal;
set work.USfinal;
  /*Exclusion*/
  if minwt<300 then delete;
  if maxwt=9999 then delete;
  if minwt=9999 then delete;
  if GA=. then delete;
  if GA=99 then delete;
  if maxwt=. then delete;
  if minwt=. then delete;
  if DELIVERY=. then DELIVERY=9;
```

```
/*Define intra-twin discordance*/
discord=100*(maxwt-minwt)/maxwt;
discord_diff=maxwt-minwt;

if discord<20 then discord_20=0;
else if discord>=20 then discord_20=1;

if discord<15 then discord_15=0;
else if discord>=15 then discord_15=1;

if discord<25 then discord_25=0;
else if discord>=25 then discord_25=1;

if GA<37 then PTB=1; else PTB=0;

if SEX=1 then GENDER=1; else GENDER=0;

if BMI>=99 then BMI=.;
if PREVIS=99 then PREVIS=.;
if PRECARE=99 then PRECARE=.;
if MEDUC=. then MEDUC=9;
if FEDUC=. then FEDUC=9;
if MRACE15=. then MRACE15=99;

/*Maternal age group*/
if MAGE<25 then MAGE_GRP=1;
else if 25<=MAGE<35 then MAGE_GRP=2;
else if MAGE>=35 then MAGE_GRP=3;

/*Mother's education group*/
if MEDUC in (1,2,3,4,5) then MEDUC_GRP=1;
else if MEDUC in (6,7,8) then MEDUC_GRP=2;
else if MEDUC=9 then MEDUC_GRP=3;

/*Mother's race group*/
if MRACE15=99 then MRACE_GRP=9; /*Unknown*/
else if MRACE15=1 then MRACE_GRP=1; /*White*/
else if MRACE15=2 then MRACE_GRP=2; /*Black*/
else if MRACE15=3 then MRACE_GRP=4; /*AIAN*/
else if 4<=MRACE15<=14 then MRACE_GRP=5; /*Asian and Pacific
Islander*/
else if 24>=MRACE15>=15 then MRACE_GRP=6; /*Mutiple race*/

/*Mother's Hispanic origin*/
if HISPANIC_M in (1,2,3,4,5) then RACE_M=3;
else RACE_M=MRACE_GRP;

/*BMI group*/
if BMI<18.5 then BMI_GRP=1;
else if 18.5<=BMI<25 then BMI_GRP=2;
else if 25<=BMI<30 then BMI_GRP=3;
```

```
else if BMI>=30 then BMI_GRP=4;
else BMI_GRP=9;

/*Weight gain tertile*/
if WTGAIN=99 then WTGAIN_GRP=9;
else if WTGAIN<30 then WTGAIN_GRP=1;
else if 30<=WTGAIN<43 then WTGAIN_GRP=2;
else if WTGAIN>=43 then WTGAIN_GRP=3;

/*Delivery method*/
if DELIVERY=9 then DELIV_GRP=9;
else if DELIVERY=4 then DELIV_GRP=1;/*Cesarean*/
else DELIV_GRP=0;/*Vaginal*/

TBO1=TBO-2;
if TBO1>0 then parity=1;else parity=0;

if WIC='Y' then WIC=1;
else if WIC='N' then WIC=0;
else WIC=9;

if RF_PDIAB='U' then RF_PDIAB=9;
else if RF_PDIAB='Y' then RF_PDIAB=1;
else if RF_PDIAB='N' then RF_PDIAB=0;

if RF_GDIAB='U' then RF_GDIAB=9;
else if RF_GDIAB='Y' then RF_GDIAB=1;
else if RF_GDIAB='N' then RF_GDIAB=0;

if RF_PHYPE='U' then RF_PHYPE=9;
else if RF_PHYPE='Y' then RF_PHYPE=1;
else if RF_PHYPE='N' then RF_PHYPE=0;

if RF_GHYPE='U' then RF_GHYPE=9;
else if RF_GHYPE='Y' then RF_GHYPE=1;
else if RF_GHYPE='N' then RF_GHYPE=0;

if RF_EHYPE='U' then RF_EHYPE=9;
else if RF_EHYPE='Y' then RF_EHYPE=1;
else if RF_EHYPE='N' then RF_EHYPE=0;

if RF_PPTERM='U' then RF_PPTERM=9;
else if RF_PPTERM='Y' then RF_PPTERM=1;
else if RF_PPTERM='N' then RF_PPTERM=0;

if RF_INFTR='U' then RF_INFTR=9;
else if RF_INFTR='Y' then RF_INFTR=1;
else if RF_INFTR='N' then RF_INFTR=0;

if RF_FEDRG='U' then RF_FEDRG=9;
else if RF_FEDRG='Y' then RF_FEDRG=1;
else if RF_FEDRG in ('N','X') then RF_FEDRG=0;
```

```
  if RF_ARTEC='U' then RF_ARTEC=9;
  else if RF_ARTEC='Y' then RF_ARTEC=1;
  else if RF_ARTEC in ('N','X') then RF_ARTEC=0;

  if RF_CESAR='U' then RF_CESAR=9;
  else if RF_CESAR='Y' then RF_CESAR=1;
  else if RF_CESAR='N' then RF_CESAR=0;

  if IP_GON='U' then IP_GON=9;
  else if IP_GON='Y' then IP_GON=1;
  else if IP_GON='N' then IP_GON=0;

  if IP_SYPH='U' then IP_SYPH=.;
  else if IP_SYPH='Y' then IP_SYPH=1;
  else if IP_SYPH='N' then IP_SYPH=0;

  if IP_CHLAM='U' then IP_CHLAM=.;
  else if IP_CHLAM='Y' then IP_CHLAM=1;
  else if IP_CHLAM='N' then IP_CHLAM=0;

  if IP_GON=1 or IP_SYPH=1 or IP_CHLAM=1 then IP_STD=1;
  else IP_STD=0;

  if IP_HEPB='U' then IP_HEPB=9;
  else if IP_HEPB='Y' then IP_HEPB=1;
  else if IP_HEPB='N' then IP_HEPB=0;

  if IP_HEPC='U' then IP_HEPC=.;
  else if IP_HEPC='Y' then IP_HEPC=1;
  else if IP_HEPC='N' then IP_HEPC=0;

  CA = CA_ANEN + CA_MNSB + CA_CCHD + CA_CDH + CA_OMPH + CA_GAST +
CA_LIMB + CA_HYPO + CA_CLEFT +
      CA_CLPAL + CA_DOWNS + CA_DISOR;
  if CA>=1 then Congenital=1; else if CA=0 then Congenital=0;
  drop CA;
run;
```
